# SMART (SiMulAtion and ReconsTruction) PET: an efficient PET simulation-reconstruction tool

**DOI:** 10.1186/s40658-018-0215-x

**Published:** 2018-09-18

**Authors:** Elisabeth Pfaehler, Johan R. De Jong, Rudi A. J. O. Dierckx, Floris H. P. van Velden, Ronald Boellaard

**Affiliations:** 1 Departments of Nuclear Medicine and Molecular Imaging, University of Groningen, University Medical Center Groningen, Groningen, The Netherlands; 2 Department of Radiology, Section of Nuclear Medicine, Leiden University Medical Center, Leiden, The Netherlands; 3 Department of Radiology and Nuclear Medicine, VU University Medical Center, Amsterdam, The Netherlands

**Keywords:** ^18^F-FDG PET/CT, Image reconstruction, PET simulation, Analytical simulation

## Abstract

**Background:**

Positron-emission tomography (PET) simulators are frequently used for development and performance evaluation of segmentation methods or quantitative uptake metrics. To date, most PET simulation tools are based on Monte Carlo simulations, which are computationally demanding. Other analytical simulation tools lack the implementation of time of flight (TOF) or resolution modelling (RM). In this study, a fast and easy-to-use PET simulation-reconstruction package, SiMulAtion and ReconsTruction (SMART)-PET, is developed and validated, which includes both TOF and RM. SMART-PET, its documentation and instructions to calibrate the tool to a specific PET/CT system are available on Zenodo.

SMART-PET allows the fast generation of 3D PET images. As input, it requires one image representing the activity distribution and one representing the corresponding CT image/attenuation map. It allows the user to adjust different parameters, such as reconstruction settings (TOF/RM), noise level or scan duration. Furthermore, a random spatial shift can be included, representing patient repositioning. To evaluate the tool, simulated images were compared with real scan data of the NEMA NU 2 image quality phantom. The scan was acquired as a 60-min list-mode scan and reconstructed with and without TOF and/or RM. For every reconstruction setting, ten statistically equivalent images, representing 30, 60, 120 and 300 s scan duration, were generated. Simulated and real-scan data were compared regarding coefficient of variation in the phantom background and activity recovery coefficients (RCs) of the spheres. Furthermore, standard deviation images of each of the ten statistically equivalent images were compared.

**Results:**

SMART-PET produces images comparable to actual phantom data. The image characteristics of simulated and real PET images varied in similar ways as function of reconstruction protocols and noise levels. The change in image noise with variation of simulated TOF settings followed the theoretically expected behaviour. RC as function of sphere size agreed within 0.3–11% between simulated and actual phantom data.

**Conclusions:**

SMART-PET allows for rapid and easy simulation of PET data. The user can change various acquisition and reconstruction settings (including RM and TOF) and noise levels. The images obtained show similar image characteristics as those seen in actual phantom data.

**Electronic supplementary material:**

The online version of this article (10.1186/s40658-018-0215-x) contains supplementary material, which is available to authorized users.

## Background

Fluorodeoxyglucose (FDG) positron emission tomography/computed tomography (PET/CT) is widely used in oncology for diagnosis of cancer, estimation of prognosis and treatment response [1–3]. PET allows the extraction of various quantitative tracer uptake metrics describing numerous phenotype characteristics of a tumour, commonly known as radiomics features, which may have added value for diagnosis or treatment follow-up [4–6]. Before these features can be used in a clinical setting, it is important to determine their repeatability and reproducibility as a function of image quality as this quality can vary by the use of different reconstruction settings and PET/CT systems [7–9].

In order to explore repeatability and reproducibility, it is necessary to analyse a large number of PET images reconstructed with different settings. Actual physical phantoms may be used for this purpose, but the collection of a large number of replicate images can be time consuming, and physical phantoms are often limited to simple geometrical shapes. Thus, to obtain a large number of images, a PET simulation tool can be useful. Such a tool would allow the fast and easy generation of a set of multiple realistic PET images using different activity distributions, acquisition settings, reconstruction settings and/or noise levels.

Several PET simulators are already available [10–19]. The most widely used PET simulators are based on Monte Carlo calculations. These calculations most accurately simulate the actual PET acquisition process and generate projection data that accurately reflect PET system data [16, 19, 20]. The projection data can then be reconstructed to generate accurate and realistic PET images. However, the main drawback of Monte Carlo simulations is that they are computationally very demanding and require a certain level of programming experience. Therefore, analytic PET simulators have also been developed with the main advantage of producing a large number of simulated images in a relatively short time [14, 15, 17]. The latter type of simulators cannot be used for detector design purposes but may be suitable for the evaluation of new image processing methods, such as automated segmentation methods, and to assess the robustness of quantitative features as function of underlying image quality, e.g. different noise levels or acquisition and reconstruction settings.

Most analytical PET simulators, which are already available, require a certain level of programming skills. The user has to get familiar with specific libraries, which makes their use difficult, especially for users with less knowledge about programming. Furthermore, to the best of our knowledge, current analytical PET simulators lack either the implementation of time of flight (TOF) or resolution modelling (point spread function, PSF). Therefore, in this work, we present SMART (SiMulAtion and ReconsTruction) PET, a rapid PET simulation and reconstruction tool that includes the implementation of three different TOF settings and two different resolution models and is easy to use. In this paper, we describe its implementation and the calibration procedure to match simulations with actual scan data. Finally, we compare results derived from simulations with those obtained from actual PET studies and compare the effect of simulated TOF changes to those expected theoretically.

## Methods

### Description of SMART-PET

**Fig. 1:**
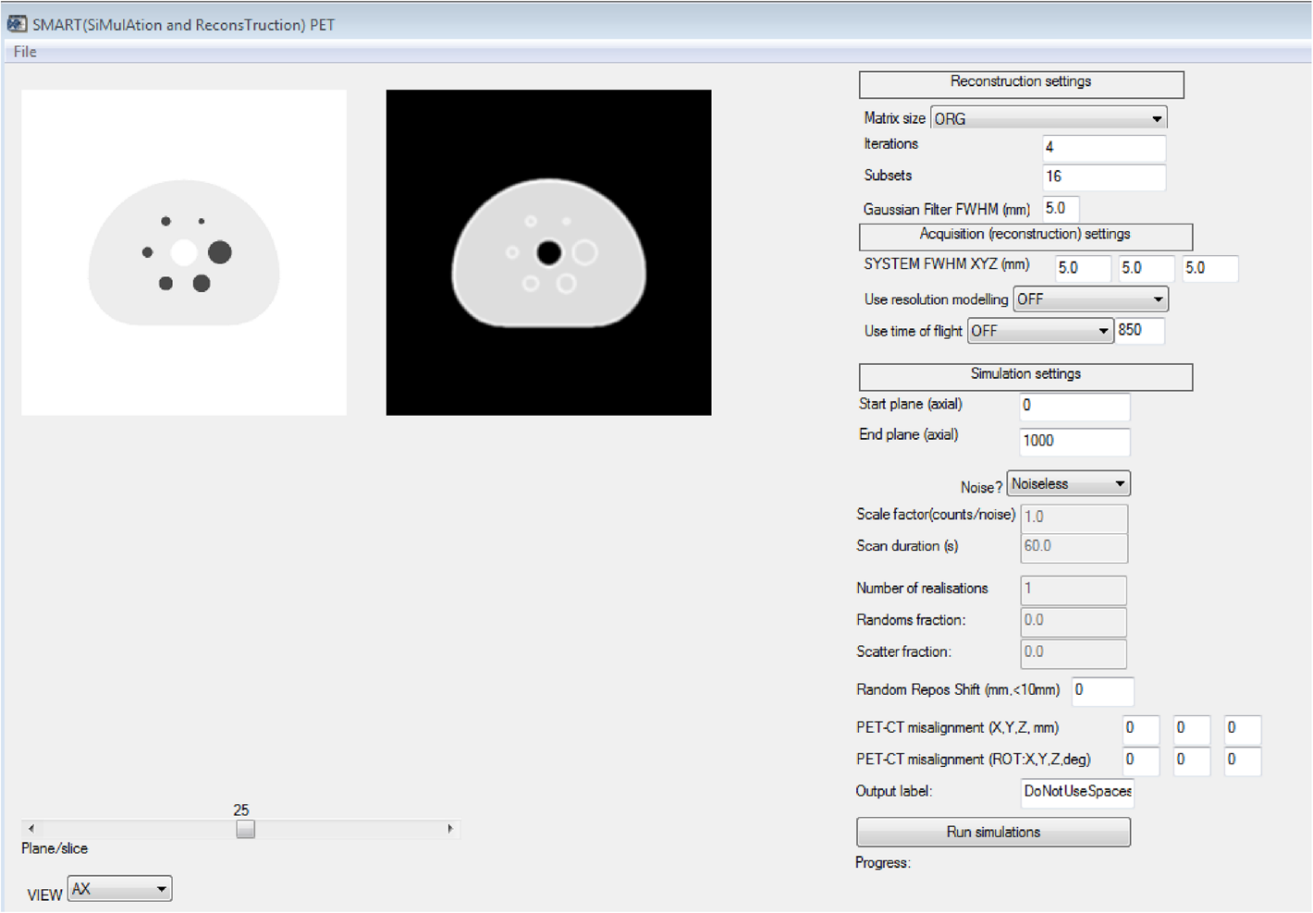
The SMART-PET GUI. On the upper left, the parameters of the reconstruction can be set, like matrix size, number iterations/subsets, resolution modelling ON/OFF and TOF ON/OFF. On the lower left, the simulation parameters like system sensitivity, random/scatter fractions and scan duration can be assigned

SMART-PET is a PET reconstruction and simulation tool that is based on analytic simulation techniques. It is a standalone program written in Interactive Data Language (IDL) (version 8.3, 64 bit; Harris Geospatial Solutions, Broomfield, CO, USA). The user can select different parameters for the simulation and reconstruction process via a Graphical User Interface (Fig. 1). A short manual on how to use and calibrate SMART-PET can also be found on Zenodo. The simulations are derived from earlier developed and applied software [21, 22]. In short, noise-free sinograms with and without TOF are generated by forward projection of a mathematical emission image. Each sinogram contains 128 angles; the number of sinogram bins is set equal to that of the matrix size of the noise-free input images (as explained later). When studying the impact of noise on image quality, multiple simulations can be created by repeatedly applying Poisson noise to the simulated noise-free sinograms. Poisson noise was applied to each sinogram and TOF bin independently using a totally random seed number. Next, the sinogram data can be reconstructed using the ordered subset expectation maximisation (OSEM) algorithm, using the attenuation correction coefficients as weighting factors. Resolution modelling can be applied during reconstruction, but a post-reconstruction iterative deconvolution approach is provided as well (Van-Cittert deconvolution). A more detailed overview of the functionality and implementation of SMART is given below.

As input, SMART-PET requires a 3D PET image representing the ‘true’ activity distribution and a 3D attenuation map or a CT image of the same object with corresponding image dimensions. At present, images should be provided in either ECAT7 or nifti format, but DICOM will be supported in future releases (work in progress).

From the input images, SMART-PET calculates a simulated PET image according to the following steps:

1. The input PET image is smoothed with a Gaussian kernel to simulate the spatial resolution of the PET camera. The full-width-at-half-maximum (FWHM, in mm) of the Gaussian kernel can be set by the user as appropriate for their system and/or in case of simulating isotopes that show a large positron range.

2. The smoothed PET image is then forward projected with or without the use of TOF. At present, TOF is applied with the number of time bins equal to the simulated image matrix size. The size of the TOF kernel (in ps) can be specified by the user. TOF is implemented by one-dimensional smoothing in the forward projection direction [23].
3. Attenuation of the PET projection data is derived from forward projecting the attenuation map and then applying an exponential function to obtain attenuation factors. In case CT data are provided, the CT image is first converted into 511 keV attenuation coefficients using a bilinear scaling function, as described by Carney et al. [24] and assuming 120 keV CT data.
4. A randoms fraction can be set. Randoms are assumed to be distributed uniformly over the projection space. The randoms fraction is defined as the fraction of simulated randoms over trues counts. This implementation has already been used in previous work [21, 25].
5. The contribution of scattered events (or the scatter fraction) can be set by the user. The distribution of scattered events is derived from the PET images by first applying a 10-cm FWHM Gaussian kernel, followed by forward projection and application of attenuation effects. The scatter fraction is defined as the total number of scattered events over true events. The implementation of scatter is analogue to the one of the analytical simulation tool PETSTEP [14].
6. Poisson noise is added to all simulated projection data. The user can define noise levels by varying the number of simulated counts and/or a noise calibration factor. Multiple noisy simulations or replicates are obtained by repeatedly drawing a noisy estimate from the Poisson distribution using the forward projected noise free counts as the expected or average value for the Poisson distribution per sinogram bin [14, 21, 26].
7. The noisy projection data are reconstructed using TOF or non-TOF ordinary Poisson ordered subset expectation maximisation (OSEM) with or without resolution modelling (applied during the reconstruction). The user can select several matrix (or voxel) sizes as well as the number of iterations and subsets.
8. A Gaussian filter and/or iterative deconvolution (using the reblurred Van-Cittert method with ten iterations [27]) can be applied upon completion of the reconstruction (post-reconstruction processing). The size of the smoothing and/or deconvolution kernel can be specified by the user.
9. Finally, the simulated image is saved in either ECAT7 or nifti format.

Steps 4 to 9 are repeated for generating a new noisy replicate. The number of desired replicates can also be set.

In addition, the user can optionally add a random spatial shift (maximum spatial displacement) to reflect variations in patient repositioning, as well as a PET-CT misalignment to simulate patient movements. The shift is applied to the emission and transmission data, while the PET-CT misalignment is only applied to the CT data. Both will be first applied in step 2, after which new noisy replicates are generated by repeating steps 2 to 9.

### Procedure to adjust SMART-PET settings to simulate your PET system

#### Calibration

The aim of the simulation tool is to produce PET images comparable to real scan data in terms of image quality and quantitative accuracy. To match the simulated PET data to the actual data of a certain PET/CT system, SMART-PET needs to be calibrated (by adjusting a sensitivity factor). To this end, the following parameters can be modified:

For the simulation step:

- System sensitivity/noise factor—this factor rescales the input data such that the simulations provide (simulated noisy) images with comparable noise as seen in the actual phantom data and can thus account for systems having different sensitivities.
- Scan duration (in seconds)
- Randoms fraction (a value between 0 and 1)
- Scatters fraction (a value between 0 and 1)

For the reconstruction step:

- Matrix and voxel size of the simulated image using either:
  - ○ ORG: The simulated image has the same voxel and matrix size as the input image
  - ○ Matrix size of 128 × 128, resulting in a voxel size of 4 × 4 mm
  - ○ Matrix size of 170 × 170, resulting in a voxel size of 3 × 3 mm
  - ○ Matrix size of 256 × 256, resulting in a voxel size of 2 × 2 mm
  - ○ Matrix size of 400 × 400, resulting in a voxel size of 1.3 × 1.3 mm

The slice thickness will not be changed and will remain equal to that of the input 3D PET image but can be varied by using an input image with a different slice thickness.

- Simulated spatial resolution of the system (default value is 5 mm)
- Reconstruction method:
  - ○ Ordinary Poisson OSEM
- TOF can be turned off (none) or set by the user.
- RM; here, the FWHM can be assigned for each direction separately (the default value is 5 mm for each direction). Any other value can be chosen to accommodate systems with a different resolution and/or when using isotopes with a larger positron range. In the case of simulating isotopes with a large positron range, the most appropriate resolution size should be chosen carefully. The resolution during simulations should be adapted to accommodate this effect on the final image resolution, as was shown by Bertolli et al. [28]. The user can either use a reconstruction-based resolution model or use a post reconstruction image-based iterative deconvolution approach (reblurred Van Cittert iterative deconvolution) [27].
- The number of iterations and subsets of the OSEM reconstruction (the default values are 4 iterations and 16 subsets).

#### Phantom scans

In order to calibrate SMART-PET, it is recommended to acquire a scan of a standard phantom (e.g. the NEMA NU 2 image quality [IQ] phantom) and reconstruct it using different reconstruction settings and with different durations/scan statistics.

As input for the simulation tool, two corresponding digital reference objects (DROs) of the same phantom are needed: One representing the activity distribution and one representing the attenuation coefficient map or CT image of the phantom. An example of a DRO of the IQ phantom is provided on Zenodo. In order to make a valid calibration, the activity distribution of the DRO and the real scan should correspond. To generate DROs with corresponding activity values, a separate DRO modifier tool, ‘IQ_DRO_Modifier’, is available on Zenodo which is also written in IDL. With this tool, the user can set the activity values in the background compartment and the spheres to the uptake values of the physical phantom data. Furthermore, the tool allows the user to change image size and slice thickness of the DROs. A short manual of this tool is provided on Zenodo. Once the DRO, which matches the actual phantom data, has been generated, a first run of simulations with acquisition and reconstruction settings that equals the standard (calibration) phantom experiment should be made. Next, by comparing the observed simulated noise with that seen in the actual phantom, the sensitivity factor can be adjusted in order to match the noise levels between simulations and real data (a higher factor represents a higher sensitivity and will result in simulated images with less percentage noise). This process may be repeated until a good agreement in noise level is observed. This factor can then be fixed, and simulations for any other conditions, acquisition and/or reconstruction settings or for other phantoms can be made. Moreover, the factor needs to be determined only once per PET/CT system.

### Validation and performance evaluation of SMART

#### Phantom experiments

In our study, the NEMA NU2 image quality phantom was scanned on a Siemens Biograph mCT 64 (Siemens Healthcare, Knoxville, USA) in order to calibrate SMART-PET and validate the accuracy of the simulated data.

The image quality phantom contains six spheres with an inner diameter of 10, 13, 17, 22, 28 and 37 mm that are placed in a background volume of 9400 ml. The spheres were filled with an FDG activity solution ten times higher than the solution in the background, which was 2.1 kBq/mL at the beginning of each PET study. The PET scan was acquired as a 60-min list mode scan. For attenuation correction purposes, a low-dose CT scan of the phantom was acquired using the vendor-provided default settings.

From the list mode data images representing 30, 60, 120, and 300 s, scan durations were generated. For every scan duration, ten statistically equal images were produced, taking into account the decay of the tracer. The data were reconstructed using the vendor-provided 3D OSEM algorithm with 3 iterations and 24 subsets. Moreover, images were reconstructed using the time-of-flight implementation of the OSEM algorithm with 3 iterations and 21 subsets. All images were generated with and without resolution modelling. All reconstructed images have a matrix size of 256 × 256 × 111 with a voxel size of 3.018 × 3.018 × 2 mm. All corrections, i.e. attenuation, scatter, random, normalisation, decay and dead time, needed to obtain quantitative PET data, were applied during the reconstructions.

#### Generation of simulated images

For the simulations, a PET and CT DRO of the image quality phantom were generated using a Matlab script (Matlab 2014b, Mathworks, Natick, MA, USA). The objects were constructed by extracting the torso shape of the phantom from a PET/CT image dataset. The spheres were then placed in the torso according to the exact details of the image quality phantom. The initial DRO PET image had a matrix size of 512 × 512 × 111 and a voxel size of 1 × 1 × 1 mm. Spheres and background intensity values were assigned in order to obtain a sphere to the background ratio of 10:1. With the DRO-modifier tool, image and voxel size as well as the activity distribution in background and spheres were matched with the actual physical phantom measurements. These digital phantoms were used as input for SMART-PET. PET images acquired with the same acquisition and reconstruction settings and scan durations as the real scan data were then produced. For every combination of reconstruction setting and scan duration, ten statistical equivalent replicates were simulated to be in line with the physical phantom experiment.

### Evaluation of SMART-PET

The simulated and physical phantom data were analysed by comparing accuracy and precision of the intensity values in the phantom background compartment and in the spheres. Furthermore, a mean and a standard deviation image of the ten replicates were calculated for each reconstruction setting and scan duration, and the standard deviation values in spheres and background were compared.

The spheres in the simulated phantom data were delineated using a Matlab-based script. Please note that Matlab scripts were only used to facilitate automated image analysis but are not part of the simulation package. Any other image analysis software may be used for this purpose as well. Six spheres with the exact radius were placed on the corresponding positions in the simulated images. For the actual physical phantom scans, the spheres were placed manually on the correct position in the phantom. Furthermore, a PET-based segmentation of the spheres was obtained using the European Association of Nuclear Medicine Research Ltd (EARL) analysis tool [29].

**Fig. 2:**
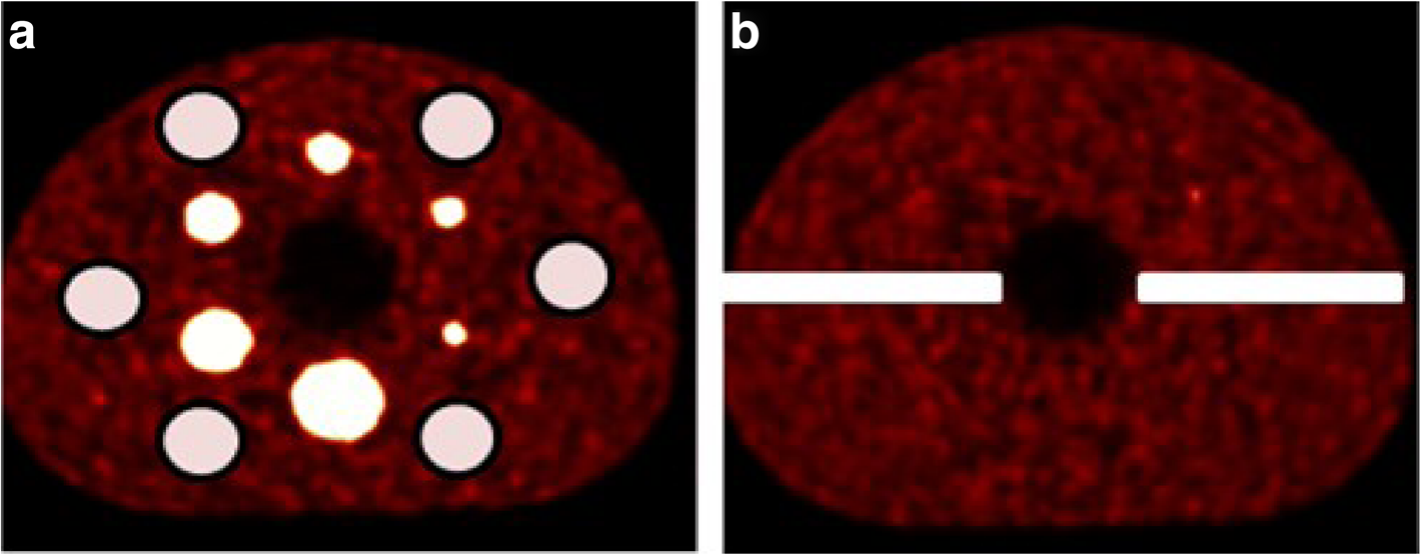
The regions of interest used to **a** calculate the COV in the phantom background at slice 38 and **b** the intensity distributions of the standard deviation image at slices 53–57 (right). Both images are generated using the physical phantom data, but the regions of interest are equally extracted from the corresponding slices of the simulated data

To compare the distribution of the intensity values in the phantom background, six spheres with a radius of 3 cm were positioned randomly in the background compartment (see Fig. 2). The overall mean intensity values of these spheres were considered to represent the average background value.

#### Coefficient of variation in the background

The coefficient of variation (COV) describes the variation in the intensity values in a region and is used here as a measure of image noise. It was calculated in the background regions of interest for every acquired image, leading to ten COV values for every combination of reconstruction setting and scan duration. For comparison, the mean of these ten values was calculated. The same analysis procedure was applied to both simulated and actual phantom data.

Furthermore, the observed COV in the simulated data was validated against the expected theoretical behaviour as function of scan duration and TOF settings. When changing the scan duration from *t*_1_ to *t*_2_, the COV is expected to follow Eq. (1):

1 $$ {\mathrm{COV}}_{t_2}=\sqrt{\frac{t_1}{t_2}\ }\ {\mathrm{COV}}_{t_1} $$

The change in COV due to the variation in TOF settings can be expected to be described by Eq. (2) [30]:

2 $$ {\mathrm{COV}}_{\mathrm{TOF}}=\sqrt{\frac{c\Delta  t}{2D}}{\mathrm{COV}}_{\mathrm{NON}-\mathrm{TOF}} $$

where *D* equals the phantom diameter, *c* the speed of light and *∆t* the TOF performance.

SMART-PET provides reconstructions with different TOF time resolutions that can be set by the user. The COV values obtained with the simulated TOF resolutions 150, 350, 450, 650 and 850 ps were compared with the theoretically expected values assuming an average phantom diameter of 27 cm.

#### Recovery coefficients

To calculate the activity concentration recovery coefficients (RC), the mean and maximum values within each sphere were calculated and divided by the expected activity concentration. RCs are plotted as function of sphere size and compared to the experimentally obtained values for each set of acquisition and reconstruction settings and for each scan duration. Furthermore, the RCs for an EARL reconstruction (OSEM + 5 mm FWHM Gaussian smoothing) and an OSEM + PSF reconstruction with 2 mm FWHM Gaussian smoothing were compared, as they are the default reconstruction settings in our department. The mean and SD of the ten statistically equivalent replicates were used for comparison.

#### Mean and standard deviation image

From the ten statistically equivalent images, a standard deviation image was calculated. In the standard deviation images, we compared the intensity values in spheres and background between simulated and physical phantom images. Furthermore, the intensity values in the centre part of the axial extend of the phantom (see Fig. 2) were extracted, and the distribution of these intensity values were plotted.

## Results

### Evaluation of SMART-PET

#### Calibration steps

To match the simulations with real scan data, the parameters of the simulation were changed in a stepwise approach. For this purpose, one scan duration and one reconstruction method was chosen. From the resulting images, the RCs and COVs in the large background compartment as well as the noise texture were compared. The simulation parameters that led to the best fit were chosen. The calibration process consists of the following steps:

*Adjusting image noise*: Several images were simulated with a noise scale factor varying from 0.001 to 0.5. The noise scale factor was set such that it provided the best fit to the physical phantom data regarding the COV in the background. Images with other reconstruction settings and scan durations were produced using this noise level scale factor.

*Matrix size*: To give a better comparison in terms of image quality and noise texture, the voxel size of the simulation was matched to the voxel size of the real scan. That led to a voxel size of 3 mm × 3 mm × 2 mm and a matrix size of 170 × 170 × 55.

*Spatial resolution of the image*: To match partial volume effects or spatial resolution, the FWHM of the system was modified. Different values, varying from 2 to 7 mm, were selected, and the RCs over the different sphere sizes were compared. FWHM of 7 mm showed the best fit with the data of the physical phantom experiments.

*Adjust iterations and subsets*: The noise texture in the simulated and physical phantom data showed a different pattern. A comparison and adjustment of the number of iterations changes this pattern and can help to match the images more closely. In our case, we selected 4 iterations and 16 subsets as default value. The Additional files 1 and 2 display simulated and physical phantom images with different numbers of iterations.

For our validation, the PET-DRO was modified so that the intensity values corresponded to an activity concentration of 23 kBq/ml in the spheres and 2.1 kBq/ml in the background compartment. The slice-thickness of the phantom was adjusted to 2 mm (equivalent to the scan).

### Comparison of COV background

**Fig. 3:**
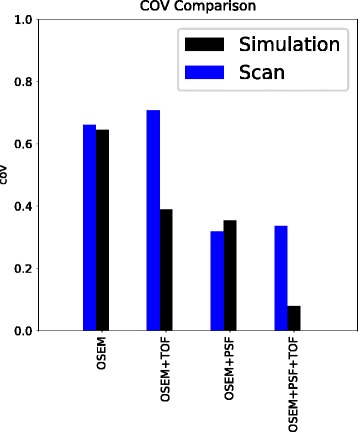
The COV values in the background compartment of simulation and scan over different reconstruction methods

**Fig. 4:**
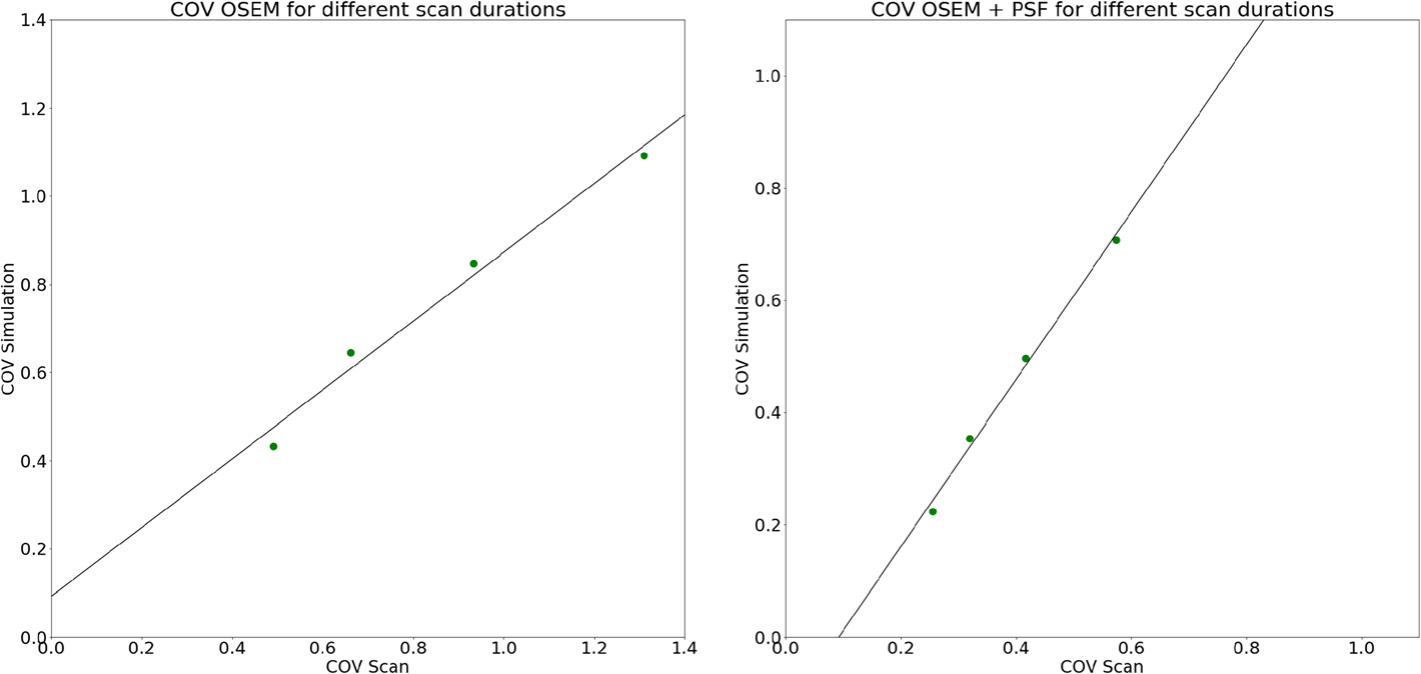
COV values in the background compartment of the NEMA image quality phantom of the simulation against the COV values of the scan for OSEM (left) and OSEM+PSF (right) reconstructions

Figure 3 shows a comparison of the COV values for simulation and phantom scans over various reconstruction settings for the 120-s scan duration. The images reconstructed with OSEM or OSEM + PSF show similar values in scan and simulation (scan: OSEM, 0.66; OSEM + PSF, 0.32; simulation, OSEM, 0.65; OSEM + PSF, 0.35). Hence, the decrease in COV from OSEM to OSEM + PSF is comparable between physical phantom (decrease of 51.5%) and simulated data (decrease of 45.3%). Thus, for these two reconstruction settings, we can conclude that the simulation follows the real scan data. As is also illustrated in Fig. 4, the values from scan and simulation are almost proportional for the different scan durations (OSEM: slope 0.78, intercept 0.09, *R*^2^ 0.99, *p* value < 0.01; OSEM + PSF: slope 1.49, intercept − 0.13, *R*^2^ 0.99, *p* value < 0.01).

Comparing the COVs over different scan durations shows that the COVs decrease with longer scan durations as expected according to formula (1) (see Table 1).

**Table 1:** The change of COV over different scan durations for the OSEM + PSF reconstruction, compared with the expected value, calculated based on the COV value at 30 s

Time (seconds)	COV simulation	Expected (acc. formula 1)
30	0.71	
60	0.50	0.50
120	0.35	0.35
300	0.22	0.22

The use of TOF in the phantom studies did not result in improved image quality (see Table 2), while it can be seen clearly in the simulations. The observed COV value in the physical phantom data (obtained with OSEM + TOF) was 78–82% higher than expected (see Table 2). This effect occurred over all scan durations. Surprisingly the physical phantom data does not follow the expected TOF behaviour, and it cannot be used for validation purposes.

**Table 2:** The differences between observed and expected improvements with the use of TOF in image quality for the physical phantom data and the simulations

	OSEM	OSEM + TOF	OSEM + TOF expected (acc. formula 2)
COV scan	0.66	0.71	0.39
COV simulation	0.65	0.39	0.39

**Fig. 5:**
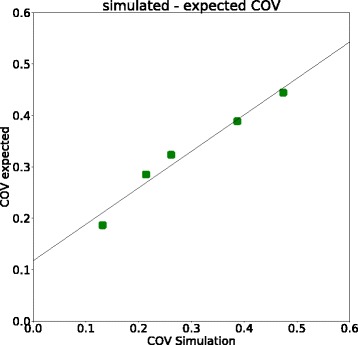
The linear relationship between the COV values of the simulated and the expected TOF values (calculated with formula 2)

Therefore, in order to validate the use of TOF in SMART, the simulated images were compared with the behaviour that would be expected theoretically (Eq. (2)). As can be seen clearly in Fig. 5, the simulated and expected COV values are showing a linear relationship (slope 0.71, intercept 0.11, *R*^2^ 0.98, *p* value < 0.01). Comparing the expected and observed SNR gains as reported in Conti et al. [31] (see Table 3) shows that the SNR gains of the simulation are similar to the ones expected.

**Table 3:** The expected and observed SNR gains for an object with 27 cm diameter

Time resolution (ps)	Expected SNR gain	Observed SNR gain in simulation
150	3.56	4.9
350	2.27	3.02
450	2.0	2.47
650	1.6	1.67
850	1.4	1.36

### Comparison of recovery coefficients

**Fig. 6:**
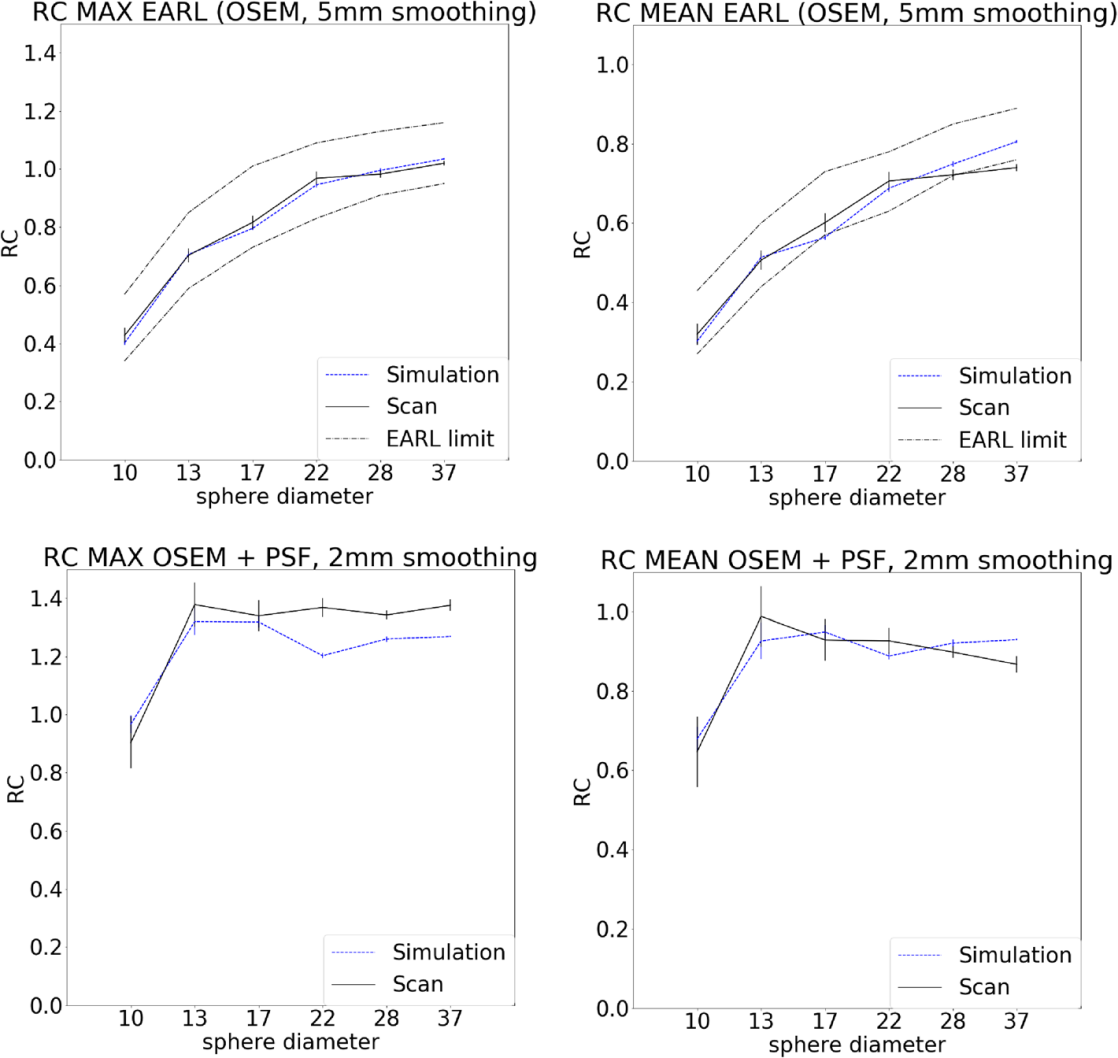
RC max (upper line) and RC mean (lower line) and the standard deviation of the RC values as function of sphere sizes for EARL reconstruction (OSEM + 5 mm smoothing) on the left and OSEM+PSF reconstruction with 2 mm smoothing on the right

Figure 6 illustrates that simulation and physical phantom studies show corresponding behaviour for RC_mean_ and RC_max_ for an EARL reconstruction (OSEM + 5 mm smoothing) with 120 s scan duration. The RCs showed an almost directly proportional relationship (RC_mean_: slope 0.87, intercept 0.07, *p* value < 0.001, *R*^2^ 0.98, RC_max_: slope 0.95, intercept 0.05, *R*^2^ 0.99, *p* value < 0.001), see also Additional files 3 and 4. Moreover, the results fulfil the EARL recommendations, as is also demonstrated in the graph. Furthermore, it can be seen that the RCs are also in line for the OSEM + PSF reconstruction with 2 mm smoothing and 120 s scan duration. The overshoot due to the Gibbs artefacts can be observed clearly. Also here, the RCs of simulation and physical phantom data show a linear relationship (RC_mean_: slope 0.79, intercept 0.19, *R*^2^ 0.92, *p* value < 0.01, RC_max_: slope 0.66, intercept 0.37, *R*^2^ 0.94, *p* value < 0.01). As the RC values for the OSEM + PSF reconstruction show few variability, a Bland-Altman plot was additionally used for comparison. This plot illustrates the low differences between the RC values of physical phantom data and simulation. Similar observations can be made for other reconstruction settings, scan durations and smoothing factors.

### Comparison of standard deviation images

**Fig. 7:**
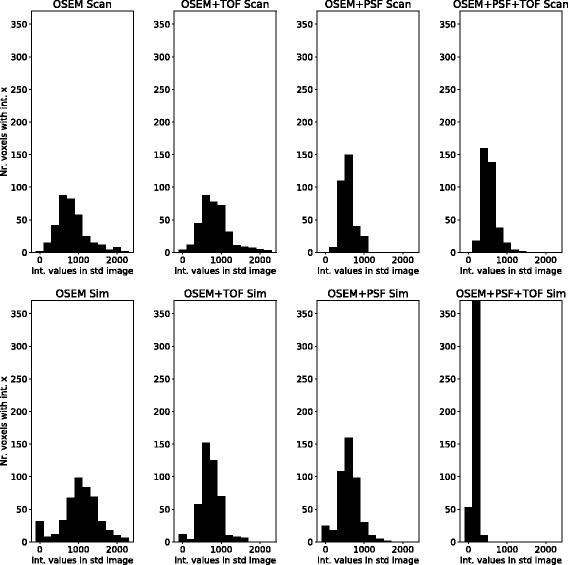
The changes in the distribution of standard deviation values over the different reconstruction settings for scan (above) and simulation (down). The values are extracted from a region in the phantom background in the standard deviation images

The distribution of the standard deviation values in the phantom background is plotted in Fig. 7. It changes for the simulations and scans in similar ways over different reconstruction settings. For the OSEM reconstruction of the simulated data, the distribution is the widest, while it becomes narrower with the use of TOF. The use of PSF leads to an even narrower distribution. The TOF effect can be seen more clearly in the simulations, which corresponds with the earlier finding that TOF does not show the (expected) increase in image quality in the real scan data. A standard deviation image of scan and simulation can be found in the supplemental (Additional file 5).

## Discussion

This paper described the development and evaluation of SMART-PET, a rapid and flexible PET simulation and reconstruction tool that allows the realistic generation of 3D PET images. It is a standalone program that can be used via a Graphical User Interface and is therefore easily accessible, especially for users with little experience with PET simulations.

SMART-PET was calibrated to the Siemens Biograph mCT 64, and we demonstrated that the simulated images show similar image characteristics as physical phantom data over different reconstruction settings and scan durations. Some discrepancies were observed when comparing the COV of the OSEM + TOF reconstructions for different TOF resolutions with the theoretically expected values. These discrepancies may be due to the fact that the expected TOF effect is calculated expecting a cylindrical object with a fixed diameter [32]. As the IQ phantom does not have a cylindrical shape, the average diameter was estimated, what can be the cause for these small differences. Furthermore, when comparing the simulated and the physical COV values over the different scan durations, the relationship between scan and simulation was not directly proportional for the OSEM + PSF reconstruction. This is due to the fact that the COVs of the physical phantom data did not follow the expected behaviour over time (according to formula 1), while the simulated data behaves as expected. However, apart from these points, we demonstrated that the images generated with SMART-PET are comparable to physical phantom data.

Depending on the matrix size and the chosen reconstruction algorithm, SMART-PET takes from 3 to 15 min to simulate one PET image. Other analytical PET simulators produce images in a comparable time. The recently developed open source software PETSTEP requires approximately 4 to 10 min for the simulation of one PET image. PETSTEP requires CERR, an open source radiology tool [33], and Matlab 2014a to produce PET simulations. It allows the simulation of PET images with manually added lesions and user-defined acquisition and reconstruction parameters. However, it lacks the implementation of TOF [14].

Another analytical PET simulator was developed by Thielemans et al. STIR is a reconstruction software that can also be used to simulate scatters, randoms and noise [15, 34, 35], as well as realistic 4D PET-MR data [18]. With STIR, realistic 3D or 4D PET images can be generated for a variety of scanners and image acquisition settings. The main drawback of simulations with STIR is the computational time required to perform a simulation (4 to 6 h for one simulation). Furthermore, the user has to be familiarised with the STIR library, which requires time and effort especially for users without comfortable use of C++.

ASIM, an analytic PET simulator developed by Comtat et al. [17] allows the simulation of static and dynamic PET images with realistic noise texture and resolution properties. The simulated projection data can be reconstructed and corrected with the same software as used in clinical practice. However, the installation and the use of ASIM requires experience in working with Linux and programming.

The most widely used PET simulator is nowadays GATE, a complex but very accurate Monte Carlo PET simulator, that allows the exact modelling of different scanner designs and detector materials [12, 36–38]. The user can accurately model the scanned object with the option to use different tissue materials and radiotracers. GATE calculates from this information a 3D PET image that includes counts, noise, randoms and scatter fractions, which fit to the modelled object and the applied radiotracer. Nevertheless, the main disadvantage of Monte Carlo simulations is the computational time cost. In order to generate accurate and correct simulations, the user has to become familiar with the GATE library, which requires time, experiences in working with the Linux operating system and basic programming. In addition, knowledge of GEANT4 is required if additional functionality needs to be implemented that is not present in GATE.

The lower computational time costs, which are coming with the use of analytical simulators, are especially of interest for studies requiring a large number of images or when modelling tracer kinetics. However, in comparison with Monte Carlo simulations, the main drawback of analytical simulators is that they lack the capability to simulate various scanner designs and detector materials. Thus, for studies investigating these parameters, SMART-PET is not suited. However, as we showed, for the exploration and evaluation of new segmentation algorithms and quantitative uptake metrics, SMART-PET produces images with good accuracy and quality.

### Limitations and future developments

In the current version of the SMART tool, some functionalities are not yet included or some approaches may be suboptimal.

First of all, the simulation tool is not yet able to simulate dynamic (kinetic) PET studies. When dynamic simulations would be required, one option would be to first make noise-free DROs per time frame, then run the simulation for each time frame independently and finally combine the simulated images into a dynamic PET study. Future releases will allow the use of a 4D dynamic DRO and automatically simulate data over all time frames. Alternatively, it may be desired to have a single DRO with per voxel specified tissue classes and assign a time activity curve to each tissue class.

Secondly, in our tool, we applied the same method to simulate scatter as was done in the PETSTEP tool [14]. Although this type of scatter simulation is based on a pragmatic choice, it may not be accurate enough in all cases and it does not include TOF information.

Furthermore, the simulation tool is lacking the implementation of Filtered-Back-Projection (FBP); as in the modern PET/CT system, mostly iterative reconstruction algorithms are used. However, we will add FBP in future releases.

In addition, at present, the resolution model used assumes spatial invariance, while in reality, the resolution of a PET/CT system varies over the field of view; in particular, it decreases at off-centre locations. This effect is mostly pronounced in systems with a small detector ring diameter, such as in dedicated brain and/or in preclinical systems.

Finally, it is of interest to expand the functionality to include more sophisticated patient motion models into the TOF-PET simulation. The approach suggested by Polycarpau et al. [39] seems interesting and should be further explored.

Despite the above listed limitations, we found that SMART seems to be suitable to generate images with realistic noise and resolution/contrast recovery characteristics that are representative for current clinical PET/CT systems.

## Conclusions

SMART-PET is a fast, easy to use and flexible PET simulation and reconstruction tool. The user can modify several parameters for the simulation and reconstruction step. In this way, SMART-PET can be adjusted to every clinical PET/CT system. As input, it requires a noiseless PET and CT image and produces a realistic PET-image that is comparable to actual PET scan data. The simulated images show similar image characteristics as real scan data. Small discrepancies between scan and simulations can be resolved by more closely matching simulation settings with the actual PET data using the described calibration process.

With SMART PET, a PET and CT mathematical phantom of the NEMA NU image quality phantom can be provided that can be used to adjust SMART-PET to a specific system. Furthermore, a DRO modifier is available that modifies the activity distribution and matrix size of the PET-DRO. The tools and DROs can be found at Zenodo.

## Additional files

Additional file 1: **Figure S1.** The behaviour of real scan data over different numbers of iterations (upper line 1–3 iterations (from left to right), lower line 4–6 iterations (from left to right)). (PDF 173 kb)

Additional file 2: **Figure S2.** The behaviour of simulated data over different numbers of iterations (upper line 1–3 iterations (from left to right), lower line 4–6 iterations (from left to right)), using 16 subsets. (PDF 237 kb)

Additional file 3: **Figure S3.** The linear relationship between the RC values of simulation and scan (RC_mean_ left, RC_max_ right). (PDF 224 kb)

Additional file 4: **Figure S4.** Bland-Altmann plot of RC_mean_ (right) and RC_max_ (left) values. The *y*-axis shows the differences between physical and simulated RC values, while the mean of these values is shown on the *x*-axis. The grey line is equal to the mean difference. (PDF 79 kb)

Additional file 5: **Figure S5.** The standard deviation image of the ten statistically images for 120 s scan duration and OSEM reconstruction (left: scan, right: simulation). (PDF 75 kb)

## Abbreviations

COV: Coefficient of variation
CT: Computed tomography
DRO: Digital reference object
FDG: Fluorodeoxyglucose
FWHM: Full-width-at-half-maximum
OSEM: Ordered subset expectation maximisation
PET: Positron emission tomography
PSF: Point spread function
RC: Recovery coefficient
SUV: Standard uptake value
TOF: Time of flight
