# Supplementary figures and images for: SMART (SiMulAtion and ReconsTruction) PET: an efficient PET simulation-reconstruction tool
Source: EJNMMI Phys. 2018 Sep 18;5:16. doi: 10.1186/s40658-018-0215-x (PMC6141406; doi:10.1186/s40658-018-0215-x)

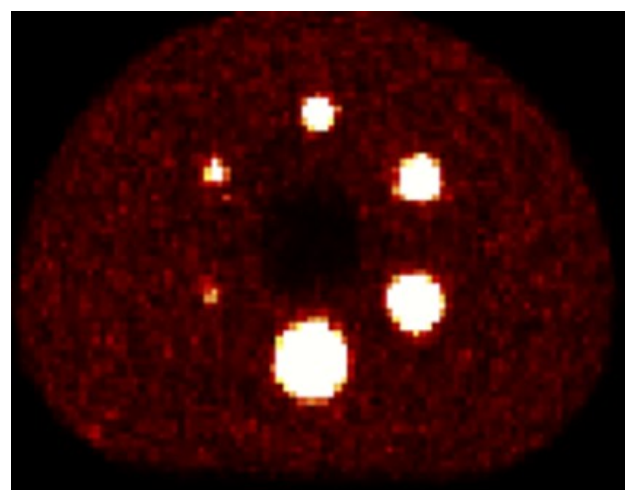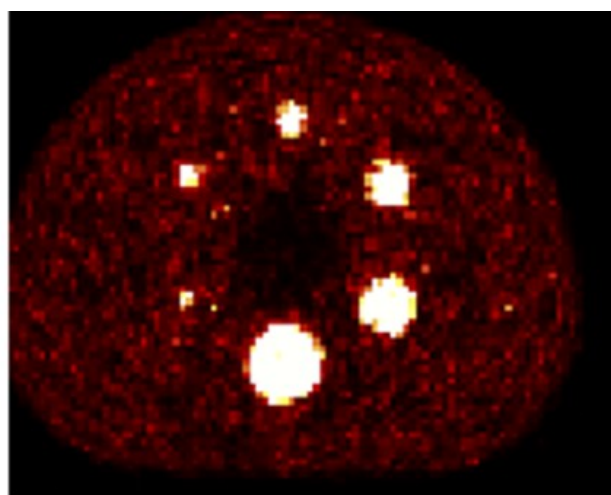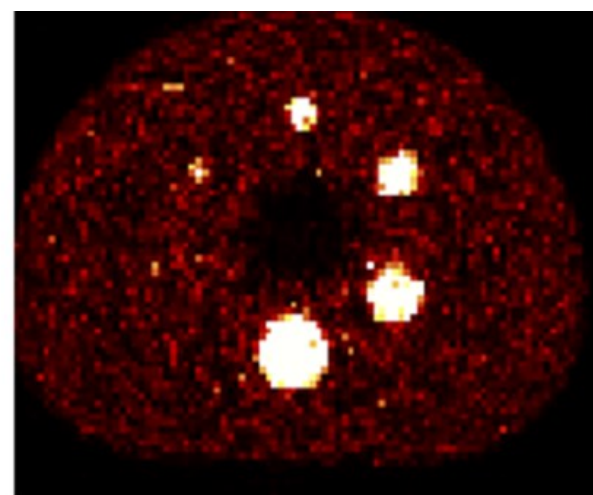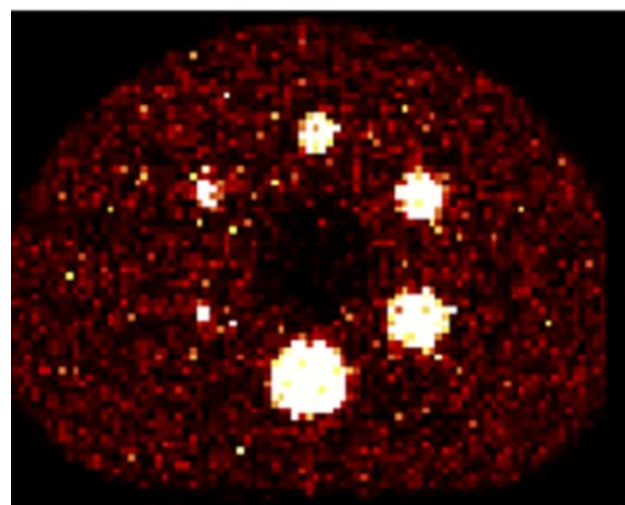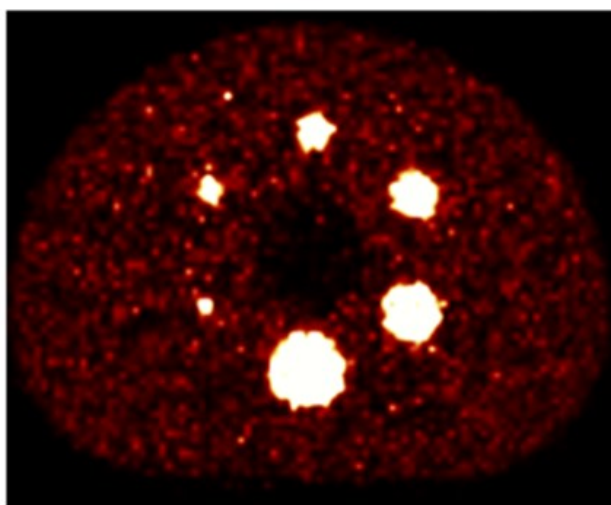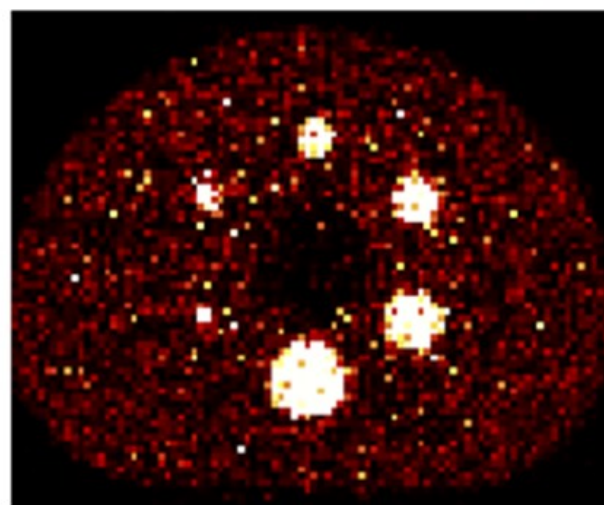

Supplement: Supplementary file 1 — Figure S1. The behaviour of real scan data over different numbers of iterations (upper line 1–3 iterations (from left to right), lower line 4–6 iterations (from left to right)). (PDF 173 kb) [file 40658_2018_215_MOESM1_ESM.pdf]

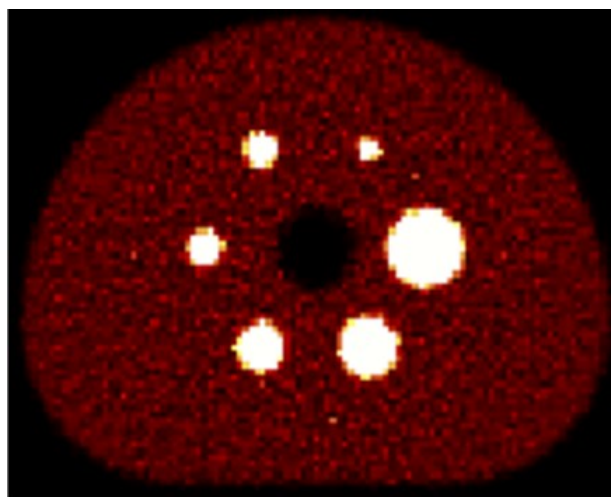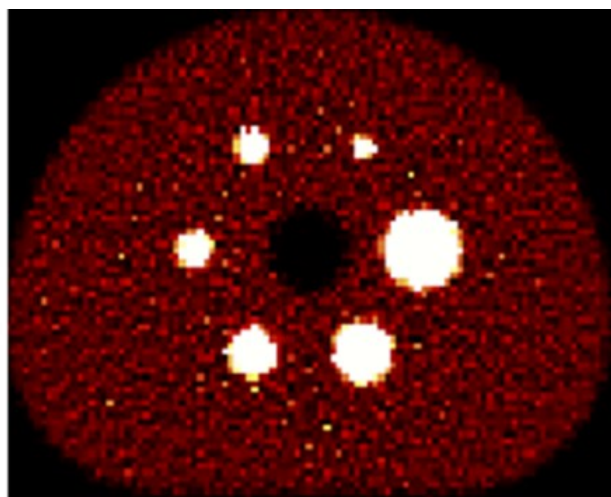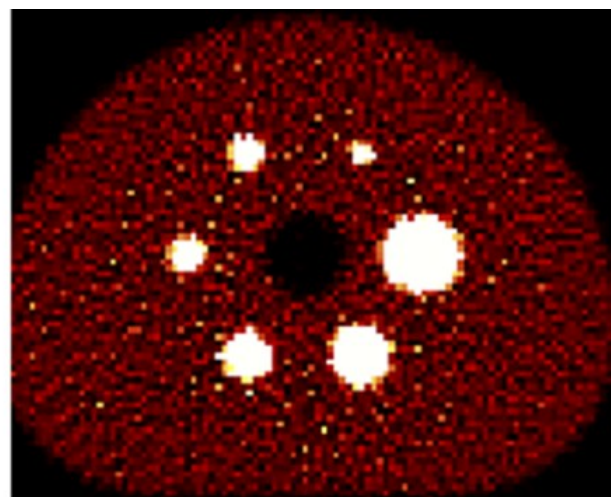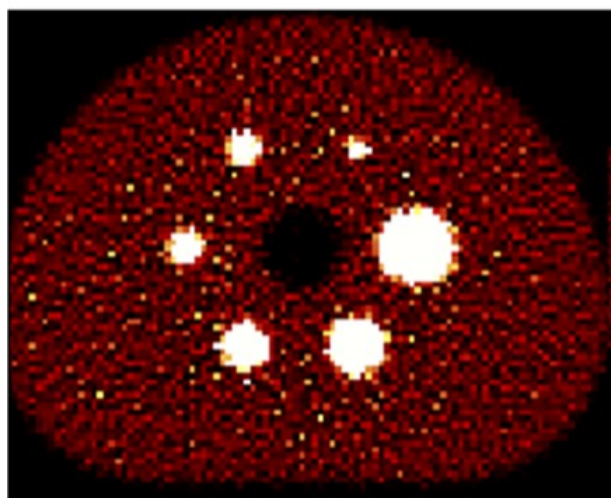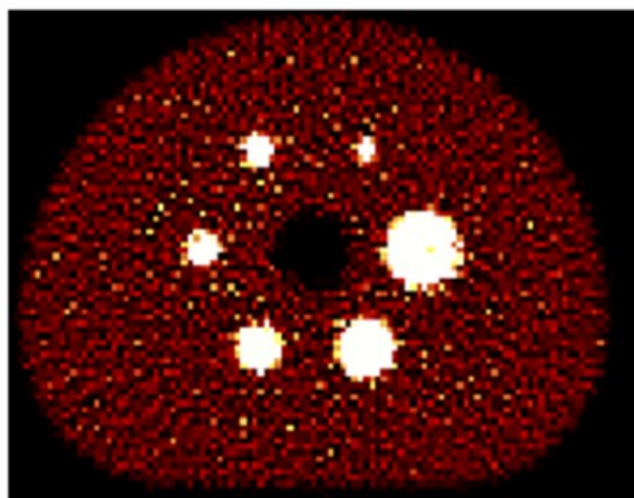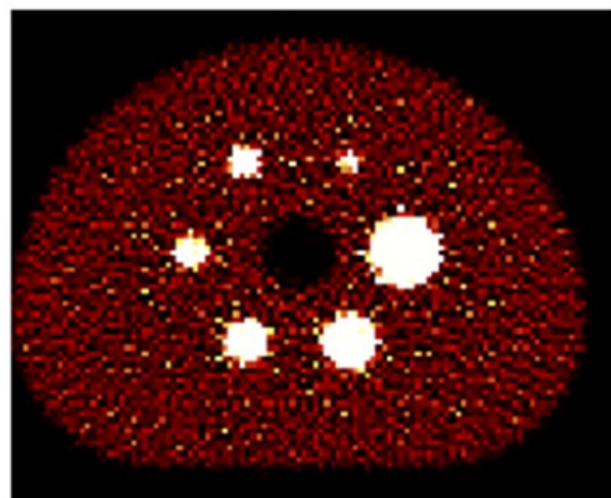

Supplement: Supplementary file 2 — Figure S2. The behaviour of simulated data over different numbers of iterations (upper line 1–3 iterations (from left to right), lower line 4–6 iterations (from left to right)), using 16 subsets. (PDF 237 kb) [file 40658_2018_215_MOESM2_ESM.pdf]

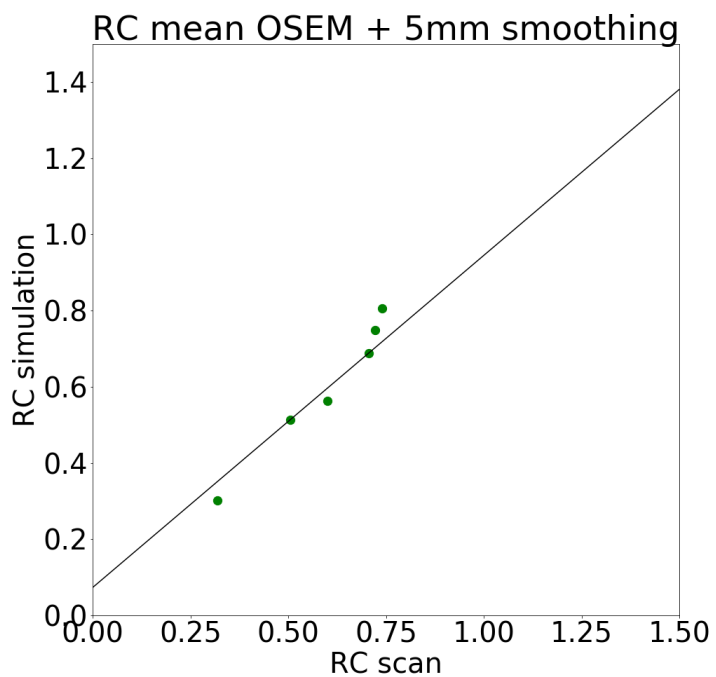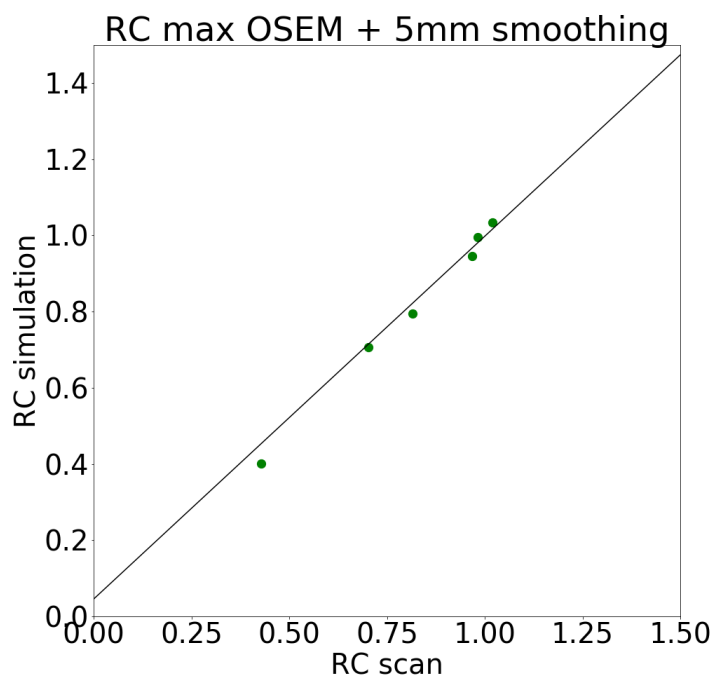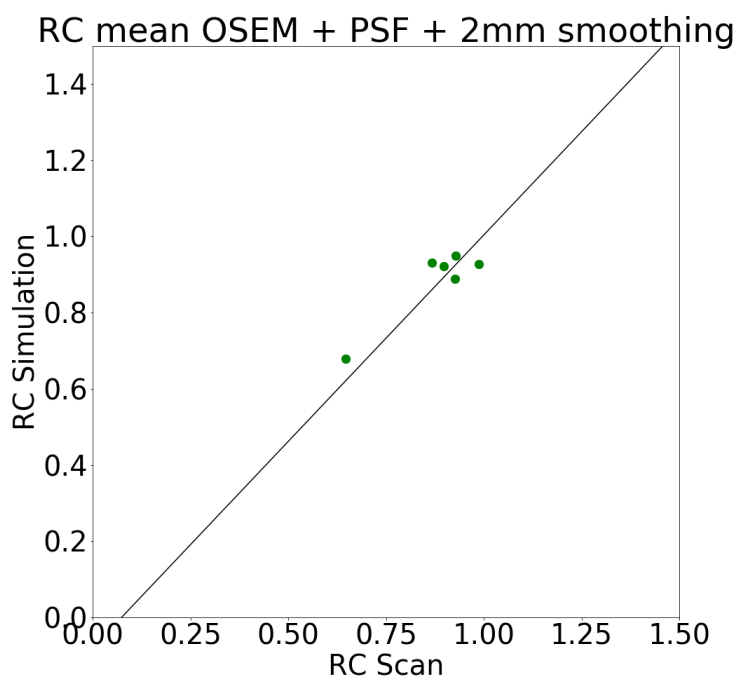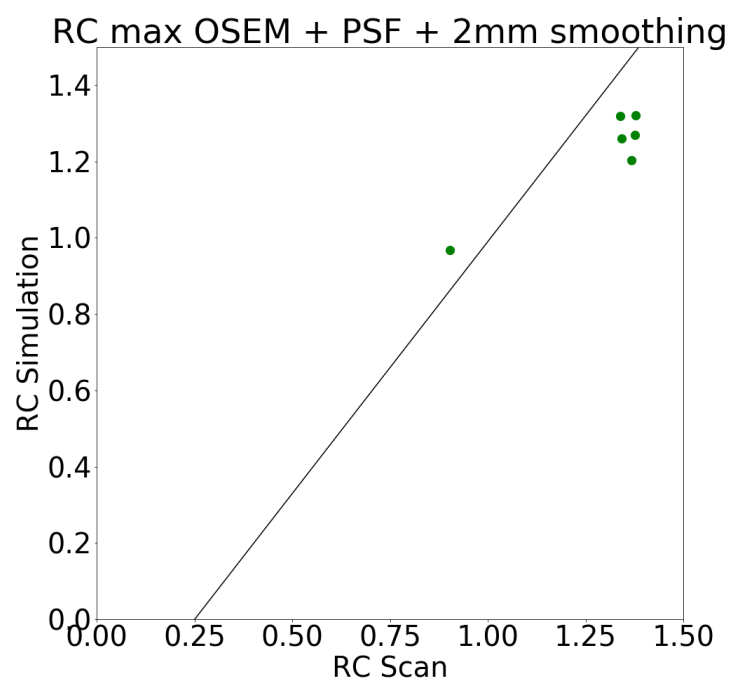

Supplement: Supplementary file 3 — Figure S3. The linear relationship between the RC values of simulation and scan (RCmean left, RCmax right). (PDF 224 kb) [file 40658_2018_215_MOESM3_ESM.pdf]

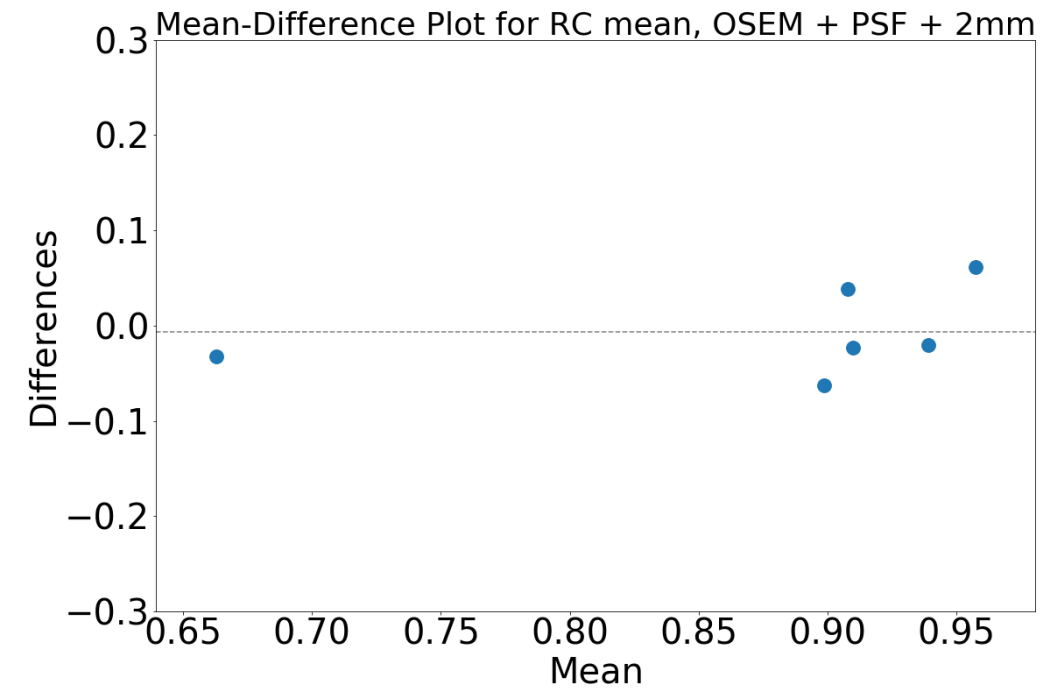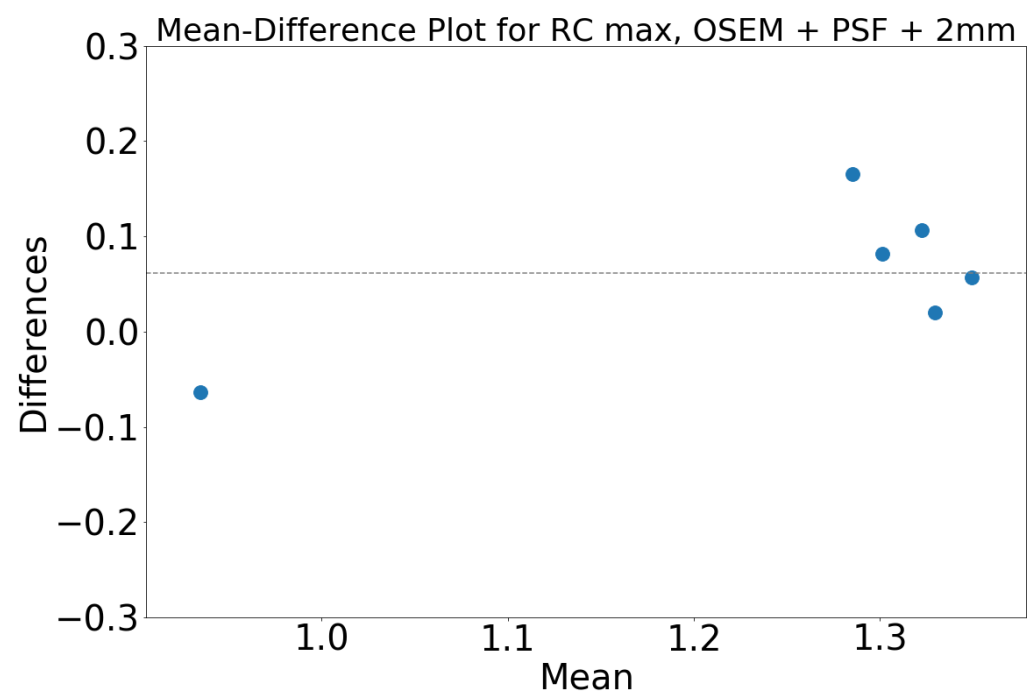

Supplement: Supplementary file 4 — Figure S4. Bland-Altmann plot of RCmean (right) and RCmax (left) values. The y-axis shows the differences between physical and simulated RC values, while the mean of these values is shown on the x-axis. The grey line is equal to the mean difference. (PDF 79 kb) [file 40658_2018_215_MOESM4_ESM.pdf]

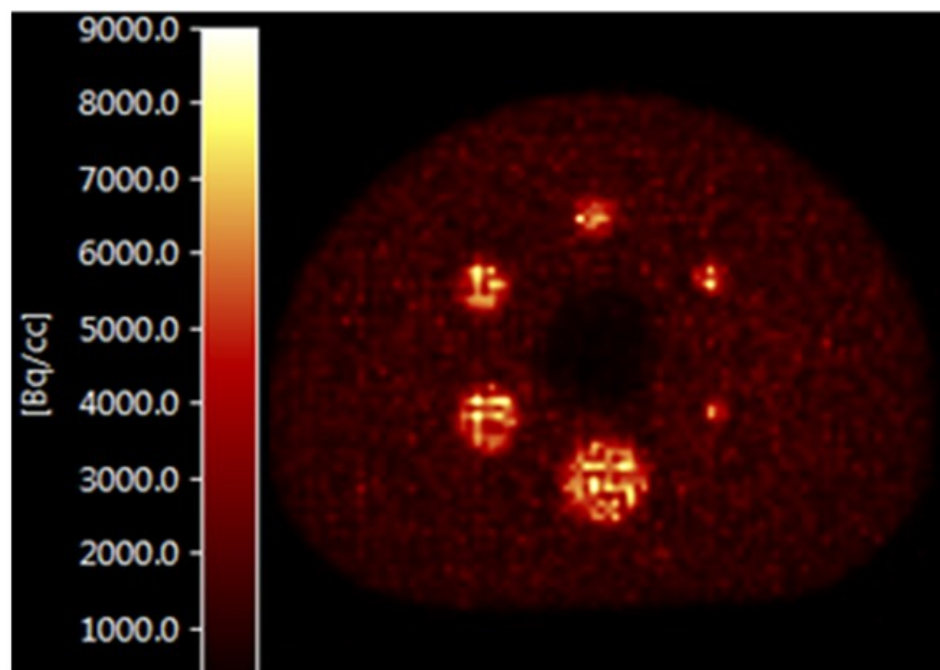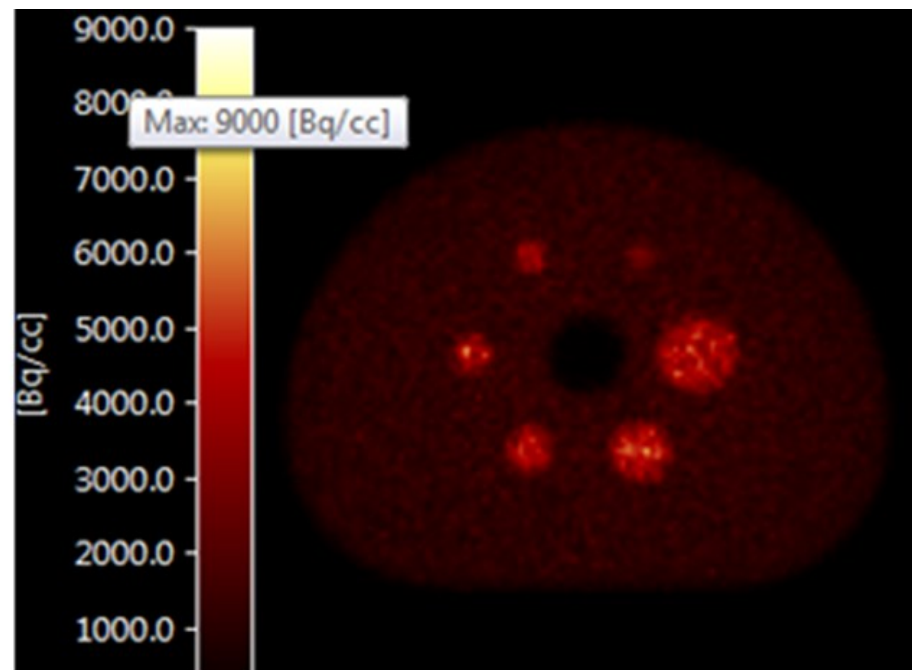

Supplement: Supplementary file 5 — Figure S5. The standard deviation image of the ten statistically images for 120 s scan duration and OSEM reconstruction (left: scan, right: simulation). (PDF 75 kb) [file 40658_2018_215_MOESM5_ESM.pdf]
